# Motivational interviewing in long-term sickness absence: study protocol of a randomized controlled trial followed by qualitative and economic studies

**DOI:** 10.1186/s12889-018-5686-0

**Published:** 2018-06-18

**Authors:** Lene Aasdahl, Vegard Stolsmo Foldal, Martin Inge Standal, Roger Hagen, Roar Johnsen, Marit Solbjør, Marius Steiro Fimland, Heidi Fossen, Chris Jensen, Gunnhild Bagøien, Vidar Halsteinli, Egil Andreas Fors

**Affiliations:** 1 Department of Public Health and Nursing, Faculty of Medicine and Health Sciences, Norwegian University of Science and Technology, Postboks 8905, MTFS, 7491 Trondheim, Norway; 2 Unicare Helsefort Rehabilitation Centre, Rissa, Norway; 3 Department of Psychology, Faculty of Social and Educational Sciences, Norwegian University of Science and Technology, Trondheim, Norway; 4 Department of Neuromedicine and Movement Science, Faculty of Medicine and Health Sciences, Norwegian University of Science and Technology, Trondheim, Norway; 5 The Norwegian Labour and Welfare Service of Trøndelag, Steinkjer, Norway; 6 National Advisory Unit on Occupational Rehabilitation, Rehabiliteringssenteret AiR, Rauland, Norway; 7 Tiller Community Mental Health Centre, Division of Psychiatry, St. Olavs Hospital, Trondheim University Hospital, Trondheim, Norway; 8 Regional Center for Health Care Improvement, St. Olavs Hospital, Trondheim University Hospital, Trondheim, Norway; 9 General Practice Research Unit, Department of Public Health and Nursing, Faculty of Medicine and Health Sciences, Norwegian University of Science and Technology, Trondheim, Norway

**Keywords:** Sick leave, Occupational health, Mental health, Musculoskeletal diseases, Case management, Return to work

## Abstract

**Background:**

Motivational interviewing (MI), mainly used and shown effective in health care (substance abuse, smoking cessation, increasing exercise and other life style changes), is a collaborative conversation (style) about change that could be useful for individuals having problems related to return to work (RTW). The aim of this paper is to describe the design of a randomized controlled trial evaluating the effect of MI on RTW among sick listed persons compared to usual care, in a social security setting.

**Methods:**

The study is a randomized controlled trial with parallel group design. Individuals between 18 and 60 years who have been sick listed for more than 7 weeks, with a current sick leave status of 50–100%, are identified in the Norwegian National Social Security System and invited to participate in the study. Exclusion criteria are no employment and pregnancy. Included participants are randomly assigned to the MI intervention or one of two control groups. The MI intervention consists of two MI sessions offered by caseworkers at the Norwegian Labor and Welfare Service (NAV), while the comparative arms consist of a usual care group and a group that receives two extra sessions without MI content (to control for attentional bias). The primary outcome measure is the total number of sickness absence days during 12 months after inclusion, obtained from national registers. Secondary outcomes include time until full sustainable return to work, health-related quality of life and mental health status. In addition, a health economic evaluation, a feasibility/process evaluation and qualitative studies will be performed as part of the study.

**Discussion:**

A previous study has suggested an effect of MI on RTW for sick listed workers with musculoskeletal complaints. The present study will evaluate the effect of MI for all sick listed workers, regardless of diagnosis. The knowledge from this study will potentially be important for policy makers, clinicians and other professionals` practical work.

**Trial registration:**

ClinicalTrials.gov: NCT03212118 (registered July 11, 2017).

## Background

Long-term sickness absence has vast consequences, not only for the society, but also for the sick-listed worker and their family [1, 2]. Hence, most industrialized countries spend considerable resources to prevent long-term sickness absence and increase the likelihood of return to work (RTW) [1]. In Norway about 5% of the gross domestic product is spent on sickness and disability benefits [3, 4].

During the last decades several interventions designed to reduce sick leave have been evaluated, but results are inconclusive [5–12]. The literature suggests that the involvement and coordination of different stakeholders are of importance [5, 9], as work disability is the result of a combination of individual, workplace, healthcare, compensation system and social factors [13]. The process of returning to work after a longer period of sick leave has been described as a dynamic process including the individual’s interactions with these factors or systems [14]. While going through this process the sick listed worker may experience different levels of self-efficacy and motivation towards behavioral change that could be necessary for RTW [14]. Sick-listed employees may improve their self-understanding and coping strategies after being challenged on self-understanding and learning through counseling from rehabilitation professionals [15]. Encounters with professional stakeholders are therefore important and special attention may be directed towards caseworkers at the Labor and Welfare Service (NAV), who administer follow-up procedures for all sick-listed citizens in Norway. Communication between sick-listed employees and caseworkers should facilitate RTW by enhancing both motivation towards behavioral change and self-efficacy. One approach which has been suggested as useful in promoting such factors is motivational interviewing (MI) [16] .

MI is a client centered and directive counselling style that elicits behavior change by helping people resolve ambivalence [17]. It was originally developed for the treatment of alcohol abuse [17], but studies have also indicated that MI is effective for other behavioral changes, for example smoking cessation [18], weight loss [19] and increasing physical activity in people with chronic conditions [20, 21]. The literature on the use of MI in RTW interventions is sparse [16, 22]. However, in a recent study comparing standard rehabilitation to rehabilitation containing MI for people sick listed due to musculoskeletal complaints, Gross et al. [23] found that participants who were employed and received MI was less likely to experience recurrence of sick leave. This suggests that MI might be useful in helping sick listed people returning to work.

The Norwegian Directorate of Health and the Norwegian Labor and Welfare Directorate first introduced MI as a counseling style to NAV, among others, through a national strategy plan for work and mental health (2007 -2012). All legal residents in Norway are included in the Norwegian public insurance system, which is administered by NAV. Medically certified sick leave is compensated 100% for up to 12 months, where the first 16 days are covered by the employer and the rest by NAV. After 12 months, it is possible to apply for more long-term benefits, which covers about 66% of the income.

In Norway, caseworkers at NAV play a central role in the follow-up of sick listed workers by coordinating RTW efforts among stakeholders and initiating (and participating) in dialogue meetings with the sick listed worker, the employer and the general practitioner. The Norwegian Labor and Welfare Directorate has suggested MI as one method that could be used to develop competence for the caseworkers when interacting with the sick listed workers. Stahl et al. [24] investigated how employees at the Swedish Social Insurance Agency experienced the introduction of MI as a method to be used in meetings with sick listed workers. However, they did not evaluate the effects of MI on RTW. Hence, there is a need to evaluate whether MI delivered by social security caseworkers can increase RTW.

Most sick-listed workers RTW in less than a month, whereas the rest has a considerable risk of persistent disability [13, 25]. Consequently, early interventions have been advocated [13, 26]. However, early intervention can delay the RTW process and hence a stepped care approach has been suggested: starting with low-intensity interventions which will be adequate for most sick listed workers, before offering more complex interventions for those who need more help to RTW [27]. MI has been suggested to be effective also when offered only as a few sessions [19, 28]; and can therefore be offered as an early low-intensity intervention.

Most RTW interventions are diagnosis specific, and mostly designed for musculoskeletal complaints [29, 30]. However, there is increasing documentation of overlap in symptoms like anxiety, depression and pain and sick-listed workers often have more than one health complaint [31–33]. Therefore, the need for diagnosis-independent interventions is starting to be recognized [34–37]. This is also in line with the paradigm shift within occupational rehabilitation, where the focus has shifted from treatment of symptoms to improvement in function [9, 13]. Since MI is a diagnosis-independent intervention, seen as a way to interact and communicate with people to strengthen their desired behaviors for RTW, its usefulness and effectiveness in people with sickness absence is therefore of great interest to explore.

### Objectives

The objective of this article is, in line with current recommendations [38], to describe the design of a randomized controlled trial (RCT) evaluating the effect of MI for sick listed workers including a health-economic evaluation, a qualitative study and a process evaluation including user perspectives and feasibility. The design of the RCT-study is three-armed, comparing 1) an intervention comprised of two MI sessions after 7 and 9 weeks of sick leave, 2) a control arm consisting of two non-MI sessions and 3) treatment as usual. The non-MI group is included to control for attentional bias from the MI- intervention. Caseworkers at social security offices (NAV) offer all the interventions. We want to explore the following research questions:

- Is MI more effective than usual care in terms of reducing sickness absence?
- Is MI cost-effective compared to usual care?
- Is MI more effective in reducing anxiety, depression and sleeping problems than usual care?
- Does the level of resilience have an impact on RTW?
- How do caseworkers at NAV and the sick listed workers evaluate the MI intervention?
- What do sick listed and caseworkers perceive as benefits and challenges in using MI to facilitate the RTW process?

## Methods/design

### Project context

This project was initiated to evaluate MI as an instrument for caseworkers at NAV in facilitating RTW for sick listed workers. Caseworkers delivered the interventions at NAV offices in Trondheim, Norway’s third largest city with 190 000 inhabitants.

### Design

**Fig. 1:**
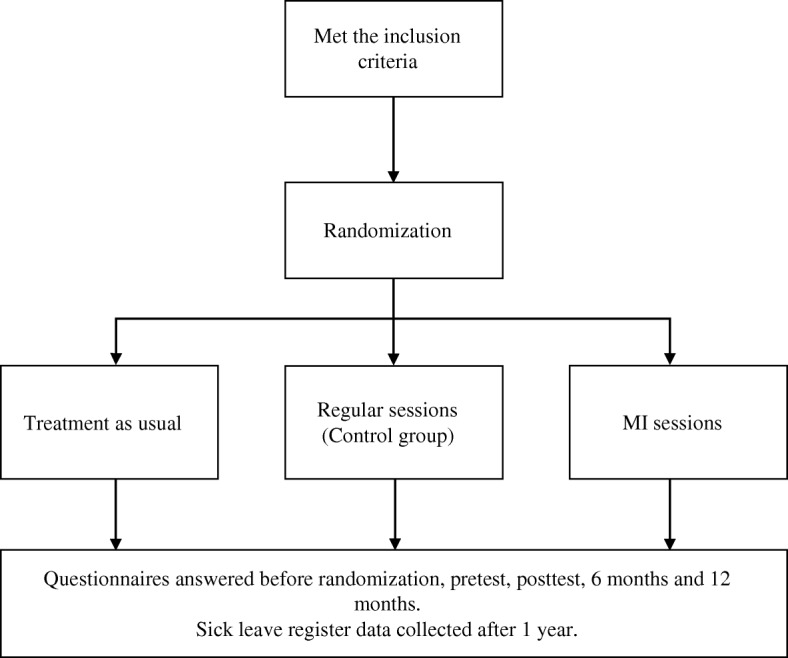
Design of the study. MI: motivational interviewing

The study was designed as a RCT with a parallel group design, followed by health-economic- and qualitative studies. Results will be reported according to the CONSORT statement, including intention-to-treat analyses [39]. The design of the study is presented in Fig. 1.

### Study population

Eligible participants are sick listed workers aged 18 to 60 years in the county of Trøndelag, belonging to NAV offices participating in this study. Participants have to be sick listed for more than 7 weeks with a current sick leave status of 50-100%, due to any diagnosis. Exclusion criteria are unemployment and pregnancy.

### Recruitment procedure for participants

NAV contacts workers sick listed for 7 weeks through their electronic communication website. This is a secure internet site where NAV communicates with individuals receiving any form of benefits. When NAV sends messages through this system, the individual receives a text message on their mobile telephone and an e-mail notifying them about the new message from NAV. Potential participants are asked to read the information about the project, accept or decline participation and sign a consent form. They reply through the electronic communication website. If they have questions, they are asked to call one of the researchers or the project’s NAV contact. If they have not replied or not read the invitation by one week, they are sent a written reminder and if they still have not answered within 3 days they are called by a NAV employee and reminded that they have a message they have not answered. A NAV employee, who is a member of the project, checks positive replies for eligibility based on the inclusion and exclusion criteria before forwarding lists of participants to the researchers.

### The interventions

*The treatment as usual group* receives the standard NAV procedure for follow-up in Norway: Within 4 weeks of sick leave, the employer and the sick listed worker must create a plan for RTW. The employer is responsible for arranging a dialogue meeting with the sick listed worker within 7 weeks of absence; other stakeholders may attend when relevant. When a worker is 100% sick listed the NAV caseworker will call the employer to support and facilitate possible work-related activities at the workplace. NAV arranges a second dialogue meeting within 26 weeks of sick leave. This meeting includes the employer and the sick listed worker, and if relevant the general practitioner. A third dialogue meeting is held if one or more of the stakeholders find it necessary [40]. In addition to the mandatory meetings, the sick listed worker may ask for meetings with NAV whenever needed. A standard RTW coordination meeting with NAV and the sick listed worker consists of a semi-structured conversation, which emphasizes the sick listed worker’s resources and needs to achieve RTW. Thus, this type of conversation is usually more instrumental in seeking solutions for RTW than motivational interviewing and for most sick listed workers it is conducted around 6 months of sick leave.

*The motivational interviewing (MI) intervention* consists of two extra sessions between the sick listed worker and a NAV-caseworker in addition to the usual NAV follow-up. The two sessions take place 7 and 9 weeks after inclusion in the study, each with a maximum length of 60 minutes. To ensure that the sessions consist of valid MI-content the NAV-caseworker uses a standardized MI-guideline developed by the research group and collaborating health care personnel with expertise on MI, in addition to input from NAV-caseworkers. The first session seeks to engage the sick listed worker in a collaborative relationship with the caseworker. Focusing the direction of the conversation by agenda mapping is included in this session as well as evoking the person’s own motivations for return to work. The first session also includes assessment of the sick listed worker according to the stages of change model, to be able to adjust the intervention accordingly to the individual [41]. The second session aims to map the sick listed individual’s work tasks, earlier attempts of RTW and RTW-self efficacy, including information exchange of possible support from NAV. In addition, the sick listed individual’s readiness to return to work is assessed. Upon completion of the two MI sessions, there are two outcomes for the sick-listed worker: Either the worker is ready for change and creates a written action plan together with NAV for RTW, or the worker decide they are not ready and no plan for RTW is made. After each session, the caseworker offer a written summary of the session.

The NAV-caseworkers involved in the MI-intervention have undergone comprehensive training in MI. Three MI-specialists (two psychologists and one psychiatrist) led this training and developed the guideline for using MI in RTW. Many of the caseworkers in the project have undergone intensive MI- training before the project started, but not all. Caseworkers without this MI-training were given 3 x 2 days workshop in MI. To assure a certain level of MI–skills, the caseworkers audio- or video-recorded a role-play using the MI guideline after the initial training, with feedback on MI-micro-skills. During the inclusion period, the caseworkers are receiving supervision from one of the MI-trainers biweekly.

*The non-MI control group* consists of two individual sessions between the sick listed worker and a NAV-caseworker in addition to usual NAV follow-up. The two extra sessions take place 7 and 9 weeks after inclusion in the study, with a maximum duration of 60 minutes. This intervention has no extra emphasis on MI, and is the standard RTW intervention clients receive in NAV when they are on long term sick leave. The caseworkers in this condition are not receiving any supervision related to their contact with their clients.

#### Randomization

Individuals who provide informed consent and are eligible will be randomized to one of the three intervention groups: MI, non-MI sessions and usual care. A web-based randomization system developed and administered by the Unit of Applied Clinical Research, Norwegian University of Science and Technology will perform block randomization.

### Effect evaluation (Randomized controlled trial)

#### Primary outcome

Total number of sickness absence days during 12 months after enrollment in the study (i.e. after randomization), obtained by national registers.

#### Secondary outcomes and additional measures

- The time until full sustainable RTW, i.e. at least 4 weeks without relapse during 12 months of follow-up, obtained by national registers.
- Probability of working (i.e. not receiving medical benefits) each month during 12 months of follow-up, measured as repeated events.
- RTW self-efficacy measured by the Return to Work Self-Efficacy Scale [42].
- Resilience measured by the Resilience Scale for Adults [43].
- Expectations about length of sick leave and return to work measured by the questions: “how long do you believe it will be until you are partly or fully back at work?” (less than 2 months, 2–4 months, 4–6 months, 6–8 months, 8–10 months, more than 10 months, I do not believe I will return to work), “How likely is it that you are back at work within 3 months?” (0–100%), “For how long do you believe you will be sick listed from today?” (free text).
- Workability measured by the single item “current workability compared with the lifetime best” from the Workability index [44].
- Health-related quality of life measured by the EQ-5D-5 L questionnaire [45].
- Pain measured by one item from the Brief Pain Inventory [46]: average pain last week on a scale from 0 to 10.
- Fatigue measured by one item from the Fatigue Severity Scale (FSS) [47]: “Fatigue interferes with my work, family or social life” (7-point Likert scale).
- Sleep problems measured by the Insomnia Severity Index [48].
- Anxiety symptoms measured by the GAD-7 [49].
- Depression symptoms measured by the PHQ-9 [50].
- Alcohol use measured by CAGE [51].

#### Data collection

Data on medical benefits will be obtained from the Norwegian National Social Security System registry, where all individuals receiving benefits in Norway are registered by their social security number. Data will be collected from two years before inclusion and up to five years after participation. Secondary outcomes are measured by self-reported web-based questionnaires at five time points: before randomization (T1), before the intervention (T2), after the intervention (T3), at 6 months (T4) and 12 months after inclusion (T5). All study-related information will be stored securely at the study site, only accessible for researchers in the project.

#### Blinding

It is not possible to blind the participants or the caseworkers. The participants provide informed consent and fill out the baseline questionnaire (T1) before treatment allocation, which is blinded. The following questionnaires are filled out at home by the participants and not likely influenced by the caseworkers. The primary outcome will be assessed by registry data and the researchers are blinded until primary outcomes are assessed.

#### Sample size

Sample size calculation was based on the primary outcome measure, number of sickness absence days. Assuming an average of 60 sickness days per year for the control group and 50 days for the intervention group (alpha 0.05, SD = 30), we would need 149 persons in each arm in order to have 80% power with a Wilcoxon rank- sum test. However, we acknowledge the fact that this power calculation is heavily based on the assumptions, and have consequently increased the sample size to 250 in each group. That should give us power to detect clinically interesting differences between the intervention groups. A statistician outside the project group performed the sample size calculation.

#### Statistical analyses

The primary outcome, number of sickness absence days, is not likely to be normally distributed and will be evaluated with a Wilcoxon rank- sum test (Mann-Whitney U-test). The analyses will be performed both as intention to treat (all randomized participants) and per protocol (participants receiving both sessions). In addition, subgroup analyses will be performed if sufficient power for age, gender, diagnoses for sick leave, occupational category and length of previous sick leave. Sustainable return to work will be evaluated with Cox regression and Kaplan Meier survival analysis with log rank test. Probability of working each month during follow-up will be measured as repeated events and analyzed with logistic General Estimating Equations (GEE). Effect differences for other secondary outcomes will be analyzed with linear mixed models and non-parametric methods, when appropriate.

### Economic evaluation

Cost-effectiveness, cost-utility and cost-benefit will be analysed in a societal perspective. Intervention costs will be calculated applying a micro-costing approach. Information about health care utilization and costs will be retrieved from national registers: The Norwegian Health Economics Administration (Helfo) and the Norwegian Patient Registry (NPR). Productivity loss due to sick leave will be calculated from number of sickness absence days combined with age and gender specific wage costs from Statistics Norway. The outcome measure in the cost-effectiveness analyses will be sickness absence days, and hence productivity costs will not be included in order to avoid double counting. In the cost-utility analyses quality adjusted life years (QALY) will be used as outcome measure, calculated by combining health related quality of life level and duration [52]. In both analyses, the incremental cost-effectiveness ratio (ICER) will be calculated by dividing the incremental cost by the incremental effect. Bootstrapping procedures will be used to estimate the uncertainty around ICER estimates. Cost-benefit analyses will compare costs and benefits between the interventions, calculating a net societal benefit.

### User perspective on the RTW process

In order to explore the lived experience of individuals on long term sick leave, we will do several qualitative studies with a descriptive phenomenological approach [53]. While surveys can map questions already known to the researchers, qualitative studies provide possibilities to identify issues that are important for study participants in their everyday lives. Exploring lived experience of sick leave and return to work includes asking questions about experiences of being on sick leave, motivation for return to work, social support and experiences of communication about being on sick leave. The qualitative studies will also explore participants’ expectations about RTW, facilitators and barriers for RTW, and the perceived benefits and challenges in using MI to facilitate a RTW-process. A nested qualitative interview study will be done among individuals who participate in the RCT. Individuals will be invited to interviews at 2 and 13 weeks after inclusion. Semi-structured interview guides are developed for each of these two lines of interviews. Recruitment will continue until data reaches enough information power [54]. Interviews will also be performed after the interventions and at follow up.

### Feasibility

In addition to evaluate the effect of an intervention, it is important to know how the intervention can be successfully implemented in daily practice. This will be evaluated by assessing implementation, accessibility and maintenance. For this evaluation, 40 cases who receive the MI intervention will be included. The degree of implementation will be measured by data from NAV on whether the intervention was carried through as planned: the number of MI sessions performed at the right time, the number of delayed and cancelled appointments. Fidelity will be assessed based on 25-30 video/audio recordings with clients. These recordings will be coded on MITI 4.2 by trained assessors outside the research group. Both quantitative (questionnaires) and qualitative data will be collected from participants and NAV-caseworkers about satisfaction with the intervention, benefits of MI as well as barriers and facilitators for implementation.

### Ethical considerations

The study is approved by the Regional Committees for Medical and Health Research Ethics in South East Norway (No: 2016/2300) and the trial is registered in clinicaltrials.gov (No: NCT03212118). All participants are given information about the study and will sign a consent form in order to be included. Regardless of which arm they are randomized to they will all receive the usual follow-up offered by NAV. Not participating in the study will have no effect on received benefits from NAV.

## Discussion

It is crucial to recognize services and structures that can help people stay at work. Returning to work after a long period of sick leave is a process where a number of factors can impact an individual’s confidence, motivation and willingness to RTW. As MI is useful when individuals are ambivalent towards behavioral change, it might also be particularly useful in the RTW context. The results of a previous study suggested an effect of MI on RTW for sick listed workers [28]. However, they only included workers with musculoskeletal disorders and recommended that future research on MI and RTW should include all sick listed workers. In this study, we will evaluate MI as an early, diagnosis-independent, low-intensity intervention offered by caseworkers at the social security office for sick listed individuals by combining an effect evaluation, an economic evaluation, a feasibility/process evaluation and qualitative studies.

A major strength, besides the RCT design, is the use of a national register for sickness absence data. This secures no recall bias and no missing data. Another strength is that all individuals who have been sick listed for 7 weeks are invited, eliminating referral bias and increasing external validity. Furthermore, besides the quantitative effect analysis, an economic evaluation and qualitative studies will be performed. In addition, the process evaluation will give information about implementation and feasibility of the intervention in practice. The knowledge from this study will be important for both policy makers, clinicians and other professionals` practical work. The main results of this study are expected to be published in 2020 in peer-reviewed journals and presented at scientific conferences.

There are some limitations to this study. As MI has been suggested explicitly as a suitable method to use within NAV, MI-training is already widespread within NAV. Hence, caseworkers offering the non-MI sessions might use content from MI. However, the caseworkers in the MI group have been given comprehensive training in MI, and will receive supervision throughout the trial and a guideline to follow during the MI session. The need for more than just initial training has been stressed in a previous study evaluating use of MI in the Swedish social security offices [24].

Eligible participants in this study are individuals who have been sick listed for at least 7 weeks. As RTW after long-term sickness absence is a process where individuals often alternate between being on and off benefits, participants might have been sick listed for more than 7 weeks the last year when counting total sickness absence. However, when retrieving registry data on sickness absence we can adjust for this in the analyses.

Another limitation is the lack of possibility to blind participants and caseworkers in this study. Furthermore, as the socio-culture and welfare systems differ considerably between countries the results from this study will not necessarily be generalizable outside this context.

## Abbreviations

MI: motivational interviewing
NAV: Norwegian Labor and Welfare Service
RCT: randomized controlled trial
RTW: return to work
